# Nutrition & Metabolism: an impressive performance since inception

**DOI:** 10.1186/1743-7075-10-1

**Published:** 2013-01-03

**Authors:** Ahmed Bakillah, M Mahmood Hussain

**Affiliations:** 1 Department of Cell Biology, SUNY Downstate Medical Center, 450 Clarkson Ave, Brooklyn, NY, 11203, USA

## 

Nutrition & Metabolism was co-founded by Drs. Feinman and Hussain in 2004 as an open access, peer-reviewed, online journal to keep readers up-to-date on recent developments in important areas of nutrition, exercise physiology, clinical investigations, and molecular and cellular biochemistry of metabolism. *The journal* is dedicated to expedite rapid publication of manuscripts that significantly advance the knowledge of nutrition and metabolism related to exercise physiology, diabetes, obesity, lipidemias, metabolic syndrome, atherosclerosis, cancer etc. *Nutrition & Metabolism* also publishes manuscripts pertaining to biochemistry of metabolism, cell signaling, molecular and cellular biology of nutrients, nutrient gene interactions and other areas that have implications for human nutrition and medicine. Current, but not exclusive, interests are metabolic effects of diet composition, interactions of macronutrients, effect of nutrients on gene expression, metabolic control and compartment models, nutritional effect of hormones, and genomic analysis of dietary phenomena.

**Figure 1:**
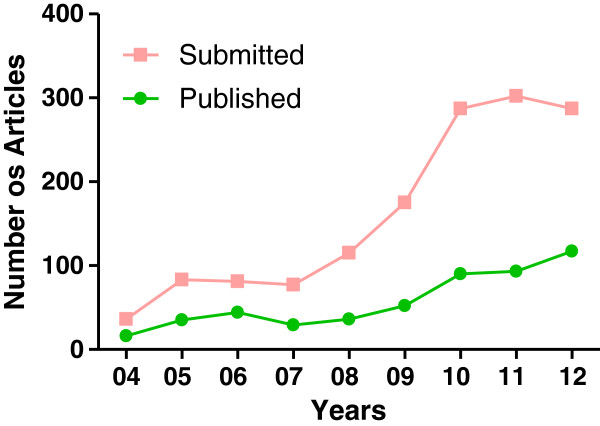
Number of articles submitted and published in the Nutrition & Metabolism (Lond) since inception.

**Figure 2:**
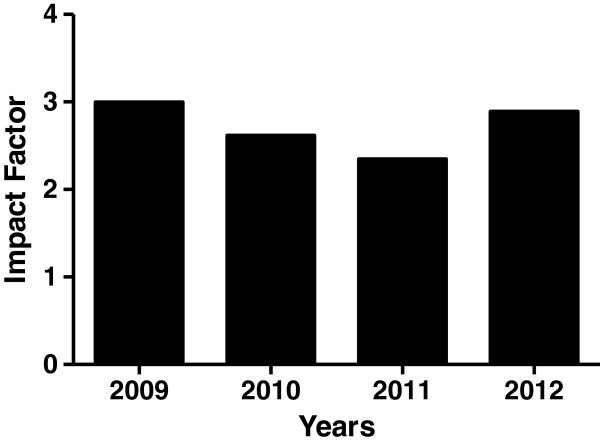
Impact Factor of Nutrition & Metabolism.

Since, its inception, the journal has seen a steady growth in the number of papers submitted (Figure 1). However, the number of papers published remained relatively small. This lead to a steady decline in the percentage of papers accepted for publication. Currently, *Nutrition & Metabolism* publishes approximately 36% of the submitted articles. The journal received an impressive first impact factor of 3.00 in 2009 indicating that published papers were highly cited soon after their publication (Figure 2). It continues to maintain its impact factor. Furthermore, several of the published papers have been highly accessed.

*Nutrition & Metabolism* publishes two main types of articles; original research articles and brief communications, although various other forms of articles such as methods, perspectives, and letters are also considered for publication. All articles must report results of well-designed research investigations that try to answer a specific hypothesis in depth using multiple approaches. Usually, the published papers provide mechanistic insights into metabolic and signaling pathways as well as clinical outcomes related to various macro- and micro-nutrients, and compounds of therapeutic potential. Manuscripts dealing with therapeutic lifestyle changes involving exercise, macronutrient content etc. are also published to provide a forum for critical, scientific evaluation of these practices or recommendations.

None of the scientific research papers are commissioned for publications. Authors elect to submit their papers for publications. Similarly, most of the reviews are submitted by investigators as they think *Nutrition & Metabolism* is the best forum for the publication of their reviews. Less than 1% of the published reviews were commissioned. The majority of solicited reviews were from editorial board members with the intention of providing guidelines to the prospective authors about the expertise of the editorial board and interest of the journal. These invited reviews were also subjected to extensive peer-review process in order to improve their quality. Hence, both invited and submitted reviews undergo extensive peer-review process.

Every paper undergoes a two-tier, quality control, evaluation process. First, Editor-in-Chief, Managing Editor, or an Associate Editors (http://www.nutritionandmetabolism.com/about/edboard) determines the suitability of the manuscript for publication in *Nutrition & Metabolism*. Articles of low perceived importance or poor quality are immediately returned to authors with no further review. If the manuscript is deemed suitable for further evaluation, it is sent for peer-review. The first priority is given to anonymous, unbiased peer-reviewers suggested by the computer based on their publications related to the subject of the article. In addition, we solicit opinion from at least one member of the editorial board. Further, consideration is also given to the reviewers recommended by the authors. Hence, every paper undergoes a stringent peer review process to evaluate objectively the quality, originality, and scientific merit of the papers. After publication, the papers are open to comments by the scientific community. The comments are first reviewed by the editors for their validity and correctness and then sent to the authors for their response. Both comments and responses are published online and have open access.

Besides the dedication and expertise of the Editorial Board members and reviewers, the success of the journal depends on the valuable contributions from investigators. Hence, we solicit comments from all the authors about the integrity and timeliness of the review process, authenticity, clarity and appropriateness of the reviews, and any other comments that would make *Nutrition & Metabolism* your first choice to disseminate your precious data.

*Nutrition & Metabolism* is published by BioMed Central, part of Springer Science+Business Media. BioMed central is committed to ensuring open access peer-reviewed biomedical research. This means that published material is freely and universally accessible online. It is archived in at least one internationally recognized free access repository, and its authors retain copyright, allowing anyone to reproduce or disseminate articles, according to the BioMed Central copyright and license agreement. *Nutrition & Metabolism*’s articles are archived in PubMed Central, the US National Library of Medicine’s full-text repository of life science literature, and also at INIST in France and in e-Depot, the National Library of the Netherlands’ digital archive of all electronic publications. The journal is also participating in the British Library’s e-journals pilot project, and plans to deposit copies of all articles with the British Library.

One of the major goals of *Nutrition & Metabolism* is the identification and publication of timely and important contributions to the field of nutrition & metabolism. We invite potential authors to visit the journal homepage http://www.nutritionandmetabolism.com, and submit their new and exciting papers online.

Ahmed Bakillah, Managing Editor

M. Mahmood Hussain, Editor-In-Chief

January 1st, 2013

